# Supplementary material for: Fabrication and Investigation of PE-SiO2@PZS Composite Separator for Lithium-Ion Batteries
Source: Materials (Basel). 2022 Jul 13;15(14):4875. doi: 10.3390/ma15144875 (PMC9322529; doi:10.3390/ma15144875)
Supplement: Supplementary file 1 [file materials-15-04875-s001.zip › materials-1724988-supplementary.pdf]

Supporting Information

Fabrication and Investigation of PE-SiO_2@PZS Composite Separator for Lithium-Ion Batteries

Liguo Xu [1,2], Yanwu Chen[1], Peijiang Liu [2,*] and Jianghua Zhan [3,*]

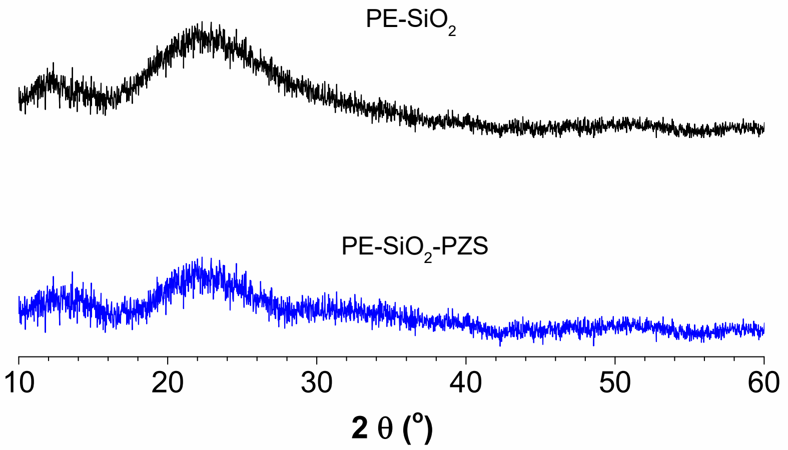

Figure S1. XRD curves for PE-SiO_2 and PE-SiO_2@PZS-1 composite membranes.
